# Leveraging Feature Fusion of Image Features and Laser Reflectance for Automated Fish Freshness Classification

**DOI:** 10.3390/s25144374

**Published:** 2025-07-12

**Authors:** Caner Balım, Nevzat Olgun, Mücahit Çalışan

**Affiliations:** 1 Department of Software Engineering, Faculty of Engineering, Afyon Kocatepe University, Afyonkarahisar 03200, Türkiye; nolgun@aku.edu.tr; 2 Department of Computer Engineering, Faculty of Engineering and Architecture, Bingöl University, Bingöl 12000, Türkiye; mcalisan@bingol.edu.tr

**Keywords:** food quality monitoring, image processing, laser reflectance, feature fusion, fish freshness classification

## Abstract

**Highlights:**

**What are the main findings?**

- A novel method for three-level fish freshness classification was developed by combining single-wavelength (940 nm) laser reflectance and deep learning-based RGB image features. This method uses a low-cost, consumer-grade laser sensor and can potentially be integrated into smartphone-level platforms in the future.
- The proposed multimodal approach achieved average accuracy of 88.44% in classifying fish freshness into three levels (Day 1, Day 2, Day 3).

**What are the implications of the main finding?**

- The fusion of laser and image data significantly improves the reliability and objectivity of freshness detection compared to single-modality approaches.
- This study provides a comprehensive dataset and an effective framework that can support future research in non-destructive food quality classification.

**Abstract:**

Fish is important for human health due to its high nutritional value. However, it is prone to spoilage due to its structural characteristics. Traditional freshness assessment methods, such as visual inspection, are subjective and prone to inconsistency. This study proposes a novel, cost-effective hybrid methodology for automated three-level fish freshness classification (Day 1, Day 2, Day 3) by integrating single-wavelength laser reflectance data with deep learning-based image features. A comprehensive dataset was created by collecting visual and laser data from 130 mackerel specimens over three consecutive days under controlled conditions. Image features were extracted using four pre-trained CNN architectures and fused with laser features to form a unified representation. The combined features were classified using SVM, MLP, and RF algorithms. The experimental results demonstrated that the proposed multimodal approach significantly outperformed single-modality methods, achieving average classification accuracy of 88.44%. This work presents an original contribution by demonstrating, for the first time, the effectiveness of combining low-cost laser sensing and deep visual features for freshness prediction, with potential for real-time mobile deployment.

## 1. Introduction

Fish is one of the most important food sources for human health. It has high levels of protein and nutrients such as omega-3 fatty acids and vitamins, which are very important for human health [1,2]. However, fish is prone to spoilage due to its structural characteristics [3]. During the spoilage process, the color of the fish’s eyeballs changes, deterioration is seen on the body surface and abdomen, muscle tone weakens, and, as a result, the nutritional value deteriorates and quality decreases [4]. The consumption of stale or spoiled fish can result not only in the loss of nutritional value and flavor but also in serious food poisoning.

There are some commonly known methods to assess the freshness of fish. Among these are methods such as examining the brightness of the skin, examining the clarity of the eyes, and checking the meat by hand. However, these methods are not very successful due to factors such as variations in visual inspection, experience level differences, and ambient light. Although new technological solutions (such as biosensors, nano-test kits) are available, fishermen and consumers do not use these services due to their limited capacity and cost [5].

The fact that fish is both economically viable and has high nutritional value has led researchers to focus on the determination of fish freshness. When the existing studies were examined, it was seen that they emphasized freshness detection and the analysis of critical parts of the fish, such as the eyes, gills, and skin surface [5,6,7,8,9]. In these studies, it was observed that convolutional neural network (CNN) techniques were mostly utilized over visual data. In addition, there are also studies in the literature on freshness detection using whole fish images [10]. There are also studies that detect freshness according to information from different sensors, such as electronic noses [11,12,13].

**Figure 1:**
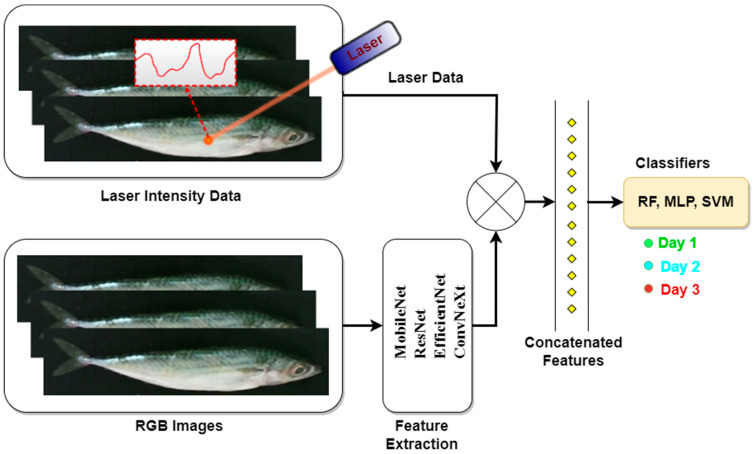
Overview of the proposed methodology integrating deep learning-based image feature extraction and laser reflectance analysis for fish freshness classification.

Many studies have confirmed that combining multiple features, instead of using a single feature, increases the validity in classification studies [14,15]. In this study, a fusion model combining 940 nm single-wavelength laser reflectance data and image features is proposed to classify fish freshness. The general structure of the study is shown in Figure 1. In the study, 130 mackerel fish were kept under controlled conditions, and 3-day images and laser data were collected for both surfaces of the fish. Deep learning models such as ResNet, MobileNet, EfficientNet, and ConvNeXt were used for feature extraction from the preprocessed images. To address the limitations of the dataset size, a transfer learning approach was adopted, in which pretrained weights from ImageNet were utilized. This allows models to effectively extract discriminative features even from relatively small datasets. A fusion approach combining image and laser data was developed to predict the freshness of the fish. These data were analyzed using support vector machine (SVM), multilayer perceptron (MLP), and random forest (RF) algorithms, and the results were comparatively evaluated using five-fold cross-validation.

In summary, the main contributions of this work include the following:

- The effects of laser data on fish freshness classification are demonstrated through detailed analyses. In addition, experimental studies demonstrate that the fusion of image and laser data performs successfully compared to single approaches.
- A comprehensive dataset including 3-day mackerel images and laser data collected systematically in 24 h periods under controlled conditions has been created for the use of researchers working in related fields.
- Feature extraction from fish images was performed using four deep learning architectures with different complexity levels, such as MobileNet, ResNet, EfficientNet, and ConvNeXt. The effectiveness of the features produced by these architectures was evaluated with SVM, MLP, and RF machine learning algorithms through extensive experimental studies.

The structure of this study is as follows. Section 2 reviews studies on fish freshness assessment methods and applications. Section 3 presents the basics of the interaction of laser beams with living tissue. Section 4 provides the details of the dataset used in the study. Section 5 presents feature extraction techniques, including the deep learning architectures, laser reflectance features, and different machine learning algorithms used in the study. Section 6 presents the results of the experiments based on multimodal data fusion, while Section 7 contains a discussion of these results. Finally, Section 8 concludes the paper with a summary of the findings and possible future directions.

## 2. Related Works

Various methods have been developed throughout history to assess fish freshness. These methods are essentially divided into two types: sensory and instrument-based assessments. In sensory evaluation, the senses of smell, taste, touch, and hearing are used by experienced people. Instrument-based assessments include chemical, biological, and physical tissue analysis, as well as spectroscopic and electrical approaches. These include biosensors, spectroscopy, and nanotechnology-based systems. In recent years, the number of image-based studies has increased, especially with the superior success of deep learning-based systems. Deep learning methods offer an important innovation in the field of fish freshness assessment, enabling faster and more precise analyses. For more information on fish freshness, the review studies in [2,13] can be consulted.

When the studies in the literature are examined, it is seen that deep learning-based image processing models have been frequently used. Some of these studies focus on the general appearance of the fish, while others focus on specific regions, such as the eyes and gills. Yasin et al. extracted features from fish images using SqueezeNet and InceptionV3 models to evaluate fish freshness and used different classification algorithms, such as SVM, ANN, LR, KNN, and RF [10]. As a result, they claimed to be able to detect fish freshness with high accuracy, ranging from 99.6% to 100%. Lanjewar and Panchbhai presented a new approach called NasNet-LSTM to predict the freshness of different types of fish [6]. In this approach, they used neural network architecture optimization, temporal dependency capture, and data balancing strategies and achieved an average F1 score of 97.1% with five-fold cross-validation.

Deep learning-based approaches focusing on the eye and gill regions are prominent in determining fish freshness. Yildiz et al. extracted features from fish eye images with the VGG19 and SqueezeNet deep learning models and presented an approach combining various machine learning algorithms (KNN, SVM, LR, RF, and ANN) to detect fish freshness [9]. As a result, they achieved accuracy of 77.3% with the combination of VGG19 and an ANN and integrated this model into a mobile application. Prasetyo et al. proposed a new CNN architecture for freshness detection from fish eye images using MobileNetV1 and depthwise separable convolution [8]. Although the proposed model is less accurate than ResNet50, they managed to provide a lightweight architecture using approximately seven times fewer parameters. Banwari et al. developed an algorithm for freshness detection from fish eye images using computer vision techniques with accuracy of 96.67% [16]. The proposed method can automatically classify the freshness level of the fish as fresh, medium fresh, and not fresh by using statistical features extracted from the HSV color space and segmentation techniques. Nguyen et al. developed an algorithm for freshness detection from fish eye images using a computer vision technique with accuracy of 96.67% [7]. The proposed method can classify the freshness level of the fish according to three classes by using statistical features extracted from the HSV color space and segmentation techniques. Akgül et al. proposed a hybrid model to automatically detect fish freshness from eye and gill colors [17]. By combining YOLO-v5 with the Inception–ResNet-v2 and Xception models, they were able to detect the freshness of anchovy and horse mackerel with high accuracy (Dataset 1: 97.67%, Dataset 2: 96.00%).

There are many studies in the literature that have evaluated fish freshness using multimodal data. Huang et al. combined computer vision and NIR spectroscopy data to provide high classification accuracy of 96.7% for Parabramis pekinensis using the BP-ANN model [18]. Kashani Zadeh et al. combined visible–near-infrared (VIS-NIR), shortwave infrared (SWIR), and fluorescence spectroscopy in a handheld device to achieve 95% accuracy [19]. Ryu and colleagues predicted freshness indicators such as pH, TVB-N, and K values in mackerel using VIS-NIR hyperspectral imaging and multivariate regression techniques (VIP-PLSR, SVR) and obtained R^2^ values of up to 91% [20]. A common feature of these studies is their reliance on data fusion obtained from multiband systems.

Unlike the studies mentioned above, this study proposes a hybrid approach to determining fish freshness by combining surface characteristics obtained from image data with optical reflection data obtained using a single-band laser reflectance module operating at a wavelength of 940 nm. The laser sensor used is low-cost and consumer-grade, operating on a principle similar to the IR-based facial recognition systems used in today’s smartphones. This similarity offers significant potential for the proposed method to be transformed into a practical freshness assessment solution that can be integrated into mobile devices in the future. Therefore, it innovatively distinguishes itself from the image and multiband spectroscopic systems in the literature in terms of optical properties, portability, cost, and tissue-focused analysis.

## 3. Laser and Tissue Interaction

**Figure 2:**
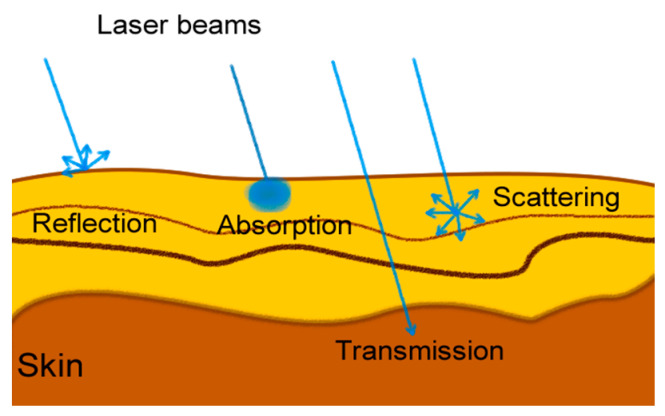
Schematic representation of the interaction of laser light with fish tissue.

The skin, which ensures the protection of living organisms from environmental factors, consists of three layers: the epidermis, dermis and hypodermis [21,22]. Fish skin has a structure that is non-keratinized, mucus-producing, and rich in collagen nets. Laser light of different wavelengths and powers can exert different effects on the tissue of living organisms. In this context, the structural properties of the skin and the interaction of light with the tissue are of great importance when analyzing the effects of laser beams on tissue. Laser beams show reflection, absorption, transmission, and scattering properties when they come into contact with tissue. Depending on the wavelength used, there may be a mixture of these. In soft tissue, the degree of reflection of laser beams between 300 and 1100 nm is 10 times higher than the degree of absorption. Figure 2 shows the interaction of laser light with tissue.

While the skin is mostly moist in the first few hours of fishing, it loses moisture in the following days and hours. While the mucus density on the skin is high at first, it decreases over time and starts to disappear. All these changes cause the hardening of the fish skin, increase the roughness of the skin, and cause changes in the absorption and reflection properties of the skin against laser light. In addition to these, as time passes, microorganisms on the surface of the fish skin multiply, causing the deterioration of the skin surface and disintegration of the skin tissue. The interaction of laser light with tissue, including variations in reflection and absorption due to morphological and biochemical changes, has also been widely studied in biomedical fields such as oncology and dentistry to identify abnormal or unhealthy tissue regions [21,23].

## 4. Dataset

**Figure 3:**
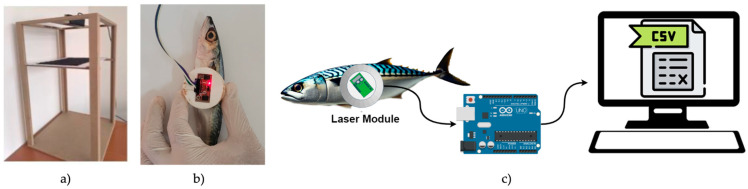
(**a**) Experimental setup for capture of fish images under controlled conditions, (**b**) laser sensor layout, and (**c**) measurement of laser reflection data from fish.

In this study, a new dataset consisting of images and laser reflection data was created to determine the level of fish freshness. In order to take fish images under controlled conditions, a special experimental setup was constructed, as shown in Figure 3a. A rectangular prism was designed for the experimental setup, and a camera was placed on its upper part to capture the fish directly. A PVC plate was placed 60 cm below the camera, and a black fabric that did not reflect light was fixed on the upper surface. The black fabric was used to create contrast in the image and to render the details of the fish clearly visible. In the imaging phase, the fish were placed on the black fabric in a fixed position, and images were taken from both surfaces. The lighting conditions were kept constant during this process.

The high-precision Time-of-Flight (ToF) TMF8821 laser sensor, manufactured by ams OSRAM AG (Tobel-bader Straße 30, 8141 Premstätten, Styria, Austria), was used to obtain fish reflection data. This sensor sends laser light to the target and provides a histogram of the number of photons reflected from the target. The ToF sensor has a 4 × 4 multizone, dynamically configurable scanning field. The sensor contains a low-power vertical cavity surface emitting laser (VCSEL) with a single wavelength of 940 nm. As shown in Figure 3b, the laser module assembly was designed in a conical shape and fixed to a 3-cm-high assembly created with a 3D printer. Figure 3c shows the measurement procedure used to obtain laser reflection data from fish. The reflection data obtained from the laser module were transferred to the computer environment with the Arduino UNO development board. Before starting the measurements, the laser sensor was configured as 3 × 3 zones. In this way, laser reflection data were obtained from nine different regions of the fish, close to each other and at the same time during each measurement. The measurement region was limited to the abdominal (ventral) mid-body area of the fish to ensure that all nine laser zones consistently fell within the body surface. Since the laser sensor captured data from adjacent zones simultaneously, narrow anatomical areas such as the eye or tail posed a risk of some beams potentially falling outside the fish.

In this study, 130 mackerel fish were used in the dataset created to evaluate fish freshness. Approximately six hours elapsed from the time that the fish were caught and then shocked, stored, purchased, and prepared for measurement. Throughout the 3-day experimental period, the fish were stored under controlled laboratory conditions at a constant temperature of approximately 4 °C. During each measurement session, fish were taken out from the cooling chamber in small groups, measured quickly, and immediately returned to cold storage. This procedure minimized exposure to external environmental factors. The relative humidity was maintained in the range of 40% to 60%, and the measurement area was arranged to prevent temperature fluctuations. Additionally, ventilation was provided to ensure stable air circulation and maintain temperature consistency.

**Figure 4:**
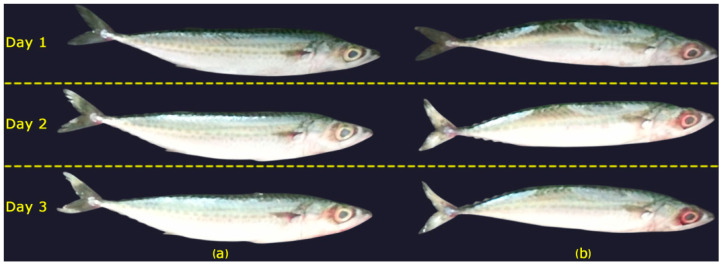
Visual changes observed in two different mackerel specimens (**a**,**b**) over a 3-day period.

Each fish was measured at 24 h intervals corresponding to Day 1, Day 2, and Day 3. Representative images illustrating the visual changes in two different mackerel samples over this period are presented in Figure 4. For each session, data were collected from both the front and back surfaces of the fish. Although the total planned number of paired samples was 780, some samples were excluded due to synchronization issues during simultaneous image and laser acquisition. As a result, two samples were removed from Day 1 and four samples each from Day 2 and Day 3, resulting in a final total of 770 matched image and laser samples. The final distribution of the dataset is presented in Table 1.

## 5. Methodology

In this study, a dataset consisting of fish images and laser reflectance values of fish bodies was created to evaluate the freshness levels of fish. Feature extraction from images was performed using deep learning models of different complexities, such as MobileNet, EfficientNet, ResNet50, and ConvNeXt. In addition to image features, laser reflectance data were used to represent the surface features of the fish. In the last stage, the extracted features were combined and fish freshness levels were classified using RF, MLP, and SVM algorithms.

### 5.1. Feature Extraction

#### 5.1.1. Image Features

In this study, CNN architectures were employed for feature extraction from images. CNNs are known for their ability to automatically learn both low-level (e.g., edges, textures) and high-level (e.g., shapes, objects) representations from images. To this end, features were extracted from fish images using four CNN-based deep learning models with varying sizes and complexities: ResNet50, MobileNetV2, EfficientNet-B2, and ConvNeXt. These models were deliberately chosen to reflect a broad range of architectural characteristics and computational requirements. ResNet50 is preferred for its high accuracy and deep residual learning capabilities. MobileNetV2 was selected for its lightweight design, which makes it suitable for resource-constrained environments. EfficientNet-B2 offers a well-balanced trade-off between performance and computational efficiency through compound scaling. ConvNeXt, on the other hand, is a modern CNN architecture inspired by Vision Transformers, while preserving the strengths of convolutional designs. A transfer learning approach was adopted using pretrained ImageNet weights, without applying any additional augmentation or normalization steps during feature extraction [24,25]. During feature extraction, the convolutional layers of all pretrained models were frozen, and no fine-tuning was applied. The input images were resized according to the input size requirements of each model: 224 × 224 pixels for ResNet50, MobileNetV2, and ConvNeXt-Base and 260 × 260 pixels for EfficientNet-B2. Brief descriptions of the models used are given below.

- ResNet50: ResNet is a CNN architecture based on blocks called “residual blocks”, developed to solve learning problems in multilayer deep networks [26]. ResNet models are named according to the total number of layers that they contain. Versions with different sizes, such as ResNet34, ResNet50, ResNet101, and ResNet152, can be selected depending on the size of the dataset and the processing resources. Although ResNet is generally superior in terms of accuracy performance, its disadvantages include long training times and high memory requirements. In this study, the medium-sized ResNet50 model was used.
- MobileNet: MobileNet is a CNN model developed for use in studies with low system requirements [27]. MobileNet is based on the “depthwise separable convolution” approach, developed to significantly reduce the computational cost used in classical convolution and to create lightweight network models. With this approach, the number of parameters and the computational cost of the model are significantly reduced. MobileNet has different versions, such as v1, v2, and v3. The MobileNetV2 version was used in this study.
- EfficientNet: EfficientNet, which has versions of different sizes between B0 and B7, is a CNN model developed to save resources with fewer parameters [28]. EfficientNet has both small- and large-scale versions: smaller models, such as EfficientNet-B0, offer low resource requirements, while large models, such as EfficientNet-B7, provide high accuracy. EfficientNet can provide similar accuracy with fewer parameters compared to models such as ResNet, making it an attractive option in terms of resource savings. The medium-sized EfficientNet-B2 model was used in this study.
- ConvNeXt: ConvNeXt is an up-to-date CNN model designed for modern computer vision tasks and was inspired by the success of Visual Transformer (ViT) architectures [29]. ConvNeXt was developed to take the simple and modular structure of ViT and combine it with CNN-specific features. In this way, significant improvements in terms of both accuracy and efficiency are achieved by utilizing the innovative structural elements of ViT, while maintaining the strengths of traditional CNNs. ConvNeXt has different versions, such as Tiny, Small, Base, and Large. The Base version was used in this study.

In the feature extraction process, global average pooling was applied to the final convolutional outputs of each CNN architecture. A fully connected layer with 128 ReLU-activated units was subsequently added to reduce the feature dimensionality. Each fish surface image was thus represented by a 128-dimensional feature vector. This uniform dimensionality across different models ensured compatibility during the multimodal fusion phase.

#### 5.1.2. Laser Reflectance Data

In this study, laser reflectance features were used to detect changes on the surfaces of the fish. For this purpose, reflectance information from nine laser channels directed at the fish body during each measurement was recorded as histograms with 128 time bins. These histograms represent the ToF distribution of photons reflected from the fish surface. Each value obtained from the histograms shows an instantaneous measurement of the interaction of the laser light with the fish tissue.

The laser sensor was fixed at a 3 cm distance from the fish surface, meaning that the earliest bins in the histogram contained reflections directly from the fish skin. In contrast, later bins predominantly capture delayed, scattered, or weak signals that often carry minimal or noisy information. An analysis of the data showed that these later bins contained high sparsity and near-zero intensity in most cases. Therefore, only the first fifteen bins from each of the nine channels were used as features, resulting in 135 laser-based descriptors per sample. This approach reduces the dimensionality while retaining the most physically and biologically relevant information for freshness assessment.

### 5.2. Feature Fusion

In this study, a feature-level fusion strategy was adopted to combine image-based features and laser reflectance data for fish freshness classification. Prior to fusion, modality-specific normalization techniques were applied to ensure scale alignment and avoid distributional bias.

Laser reflectance features were normalized using z-score normalization, which centers the data by subtracting the mean and scales them via the standard deviation. This approach is effective in handling sensor-based data with varying dynamic ranges.

Image features extracted via pretrained CNN models (ResNet50, EfficientNet, ConvNeXt) were normalized using min–max normalization, which rescales feature values into the [0, 1] range, preserving the relative distances and enhancing the compatibility with the classifier input range.

Following normalization, the two feature vectors were concatenated into a single unified representation, which enabled the joint use of both modalities in subsequent classification tasks. This approach, classified as early fusion, makes use of the complementary strengths of each data type. While the laser data captures information about the physical surface structure, the image data provide valuable visual appearance cues. Together, they contribute to a more comprehensive understanding for the classification process.

### 5.3. Machine Learning Algorithms

#### 5.3.1. Random Forest

RF is a powerful ensemble learning algorithm, widely used in both classification and regression problems [30]. RF aims to obtain more stable and accurate results by combining the predictions of multiple decision trees. RF has the advantages of being able to determine feature importance levels, handle missing data effectively, and perform parallel computation.

#### 5.3.2. Support Vector Machine

SVM is a powerful supervised learning algorithm, widely used in machine learning [31]. SVM aims to maximize the margin between classes by finding separating planes between data points. Thanks to its kernel functions, SVM can move nonlinear data into a high-dimensional space and make them separable from each other. This feature has made SVM a widely used algorithm in nonlinear classification problems.

#### 5.3.3. Multilayer Perceptron

An MLP is a feedforward artificial neural network model, widely used in machine learning and deep learning [32]. An MLP has a structure consisting of an input layer, one or more hidden layers, and an output layer. Each layer consists of neurons connected to each other with different weights. MLPs can model nonlinear relationships using activation functions such as ReLU and Sigmoid. In an MLP, at each epoch, the error between the output of the network and the target values is calculated by a backpropagation algorithm, and the error is minimized by using gradient descent optimization, etc. The MLP, which can be used in both classification and regression tasks, provides successful results, especially for multidimensional data.

### 5.4. Performance Metrics

To evaluate the effectiveness of the proposed method, four standard metrics, namely accuracy, precision, recall, and F1, were used. Table 2 details these metrics and their formulas. In the table, TP represents true positive predictions, TN represents true negative predictions, FP represents false positive predictions, and FN represents false negative predictions.

## 6. Experiments

### 6.1. Experimental Details

In this study, the performance of each classifier was evaluated using different combinations of image- and laser-based features through five-fold stratified cross-validation. For each fold, the dataset was randomly partitioned into 80% training and 20% testing subsets. Image features were extracted using four pretrained CNN architectures: ResNet50, MobileNetV2, EfficientNet-B2, and ConvNeXt. Each fish surface image was represented by a 128-dimensional feature vector. Similarly, laser reflectance data were transformed into a 135-dimensional feature vector for each sample. These image and laser features were then concatenated to form a unified 263-dimensional multimodal representation. Classification was carried out using SVM, RF, and MLP algorithms. The hyperparameter settings used in the classifiers are summarized in Table 3. All data processing, feature extraction, and classification were performed using Python 3.10 (TensorFlow, Scikit-learn) on a Linux-based system.

### 6.2. Experimental Results

In this study, fish freshness classification was performed on a daily basis with three different classifiers using visual and laser data. The results obtained from the experimental studies are shown in the tables below. The most successful results are shown in bold.

Table 4 shows the classification results with the SVM, RF, and MLP algorithms using only data from laser sensors. The results show that the accuracy of the laser data is relatively low. RF was the most successful algorithm and outperformed the other algorithms, with average accuracy of 69.22%. This indicates that laser data alone have limited predictive capacity.

Features were extracted from the visual data using four pretrained CNN (ResNet, EfficientNet, MobileNet, and ConvNeXt) models, and classification was performed with the SVM, RF, and MLP machine learning algorithms. The results obtained are shown in Table 5. When the results are analyzed, it is seen that the combination of features obtained with ConvNeXt and the SVM classifier performs best, with average accuracy of 85.97%. These results show that ConvNeXt provides a stronger representation than the others in the extraction of visual features, and SVM is effective in classification. The second-best performance was obtained by classifying the features extracted by the ResNet50 model using the MLP, achieving average accuracy of 84.29%.

The experimental results obtained by using laser data and visual features together are given in Table 6. The combination of visual features and laser data provided higher accuracy rates in the experiments. The most successful results were obtained with an average accuracy rate of 88.44% when the features extracted from the ResNet50 model and the data obtained from the laser sensors were used together and classified with the MLP. This shows that laser and visual data provide complementary information and improve the prediction performance when used together. The second most successful result was obtained with an average accuracy rate of 88.31% in the SVM classifier with the data obtained from the laser sensors and the features extracted from the ConvNeXt model.

**Figure 5:**
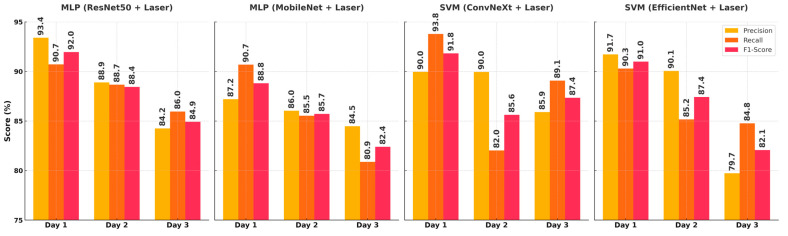
Comparison of performance metrics by day for classifiers that give the best results among all models combining image and laser features.

Figure 5 presents the precision, recall, and F1-score values for four different model–feature combinations across three days. The best overall performance was achieved by the ResNet50 + Laser + MLP model on Day 1, with precision of 93.41% and an F1-score of 91.96%, indicating highly accurate and balanced predictions. The ConvNeXt + Laser + SVM combination reached the highest recall value of 93.79% on the same day, highlighting its ability to detect fresh samples without missing relevant instances. While the MobileNet and EfficientNet models also demonstrated relatively strong performance on Day 1, a decline was observed on Days 2 and 3, particularly in the F1-score. Although the recall values were relatively preserved on Day 2, both the precision and F1-score showed a general decrease. By Day 3, all models exhibited more pronounced drops, especially in the recall and F1-scores, with the lowest F1-score of 82.07% recorded by the EfficientNet + SVM model. These results suggest that discriminative features related to fish freshness become less distinguishable over time, and the most reliable classification performance is generally obtained on the first day.

## 7. Discussion

In this study, a method for fish freshness classification was developed by combining laser data and image features. The results of the experiments provide a detailed description of the performance of the different types of data and machine learning algorithms used. The results obtained with only laser data show that this type of feature has limited predictive capacity. Although the RF algorithm performs the best, with accuracy of 69.22%, the overall accuracy is low, indicating that laser reflectance data alone are not sufficient.

In the experiments using only images, the combination of ConvNeXt features and SVM provided the best results, with average accuracy of 85.97%, while the combination of ResNet50 features and the MLP performed similarly, with average accuracy of 84.29%. These findings suggest that visual features provide stronger discriminative information for freshness prediction. In the experiments where laser reflectance data and visual data were combined, the performance improved significantly, with the MLP classifier achieving the highest performance, with average accuracy of 88.44%. This shows that data from laser sensors can be effective in providing complementary information for freshness prediction, but other types of data are needed for robust predictions. In particular, the visual features extracted by CNN models, such as edges, textures, and colors, are complemented by the reflectance data from laser sensors, demonstrating that combining these two data sources strengthens class separation and improves the overall model performance.

It is crucial to evaluate the success metrics used in classification problems not only in statistical terms but also in the context of applications. As in this study, misclassifications in classification problems related to food safety can lead to different outcomes; for example, misclassifications can increase public health risks, cause economic losses, or negatively affect the supply chain management efficiency. Therefore, a high recall value in the Day 3 class, i.e., the rate at which examples belonging to this class are correctly recognized, is critical for the reliability of the system. On the other hand, a high precision value in the Day 1 class is important to prevent examples belonging to this class from being incorrectly eliminated. The F1-score, which optimizes both metrics together, should be preferred as a balanced and comprehensive success indicator across all classes.

The results presented in Figure 5 demonstrate that the classification performance should be evaluated not only in statistical terms but also in the context of applications. In particular, in the Day 1 class, the ResNet50 + Laser + MLP model shows that examples belonging to this class are recognized with high accuracy and that the misclassification rate can be kept low, with a precision value of 93.4% and an F1-score value of 91.9%. The ConvNeXt + Laser + SVM model, on the other hand, was the most successful combination in accurately capturing Day 1 samples, with a recall value of 93.8%. This is important in terms of maintaining product quality in the supply chain and presentation to the consumer. However, a decrease in the precision and recall values was observed in all models in the Day 2 class. The intermediate transition feature of this class and its similarity to other classes in terms of both visual and optical characteristics resulted in limited discriminative power. In the Day 3 class, the recall values of some models being above 90% indicates that the samples belonging to this class are largely correctly identified and supports the functionality of the system in terms of food safety. All these results demonstrate that the F1-score is a suitable performance metric for model comparisons and reliability assessments by balancing precision and recall.

**Figure 6:**
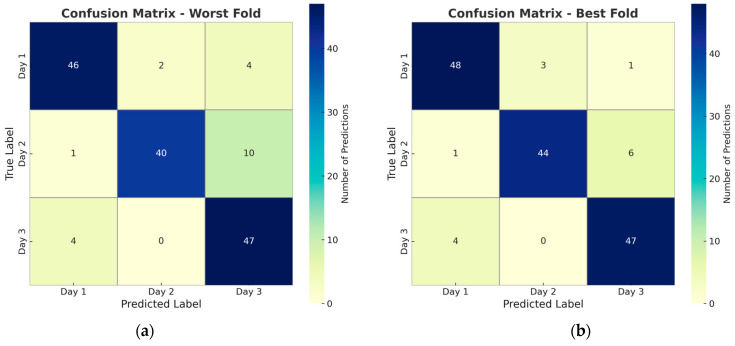
Confusion matrices for ResNet50 + MLP model in (**a**) the worst-performing fold and (**b**) the best-performing fold of the five-fold cross-validation.

The confusion matrices of the lowest (a) and highest (b) classification results of the ResNet50 + MLP model, which is the most successful model, are shown in Figure 6. When examining the confusion matrices, it was observed that the most successful model, the ResNet50 + MLP combination, yielded accurate predictions in the Day 1 and Day 3 classes, but its errors increased in the Day 2 examples. In particular, the classification of some Day 2 examples as Day 3 shows that the model has difficulty in determining the transition from freshness to deterioration. This situation stems from the transitional nature of the Day 2 class and the high visual and physical similarity between these two classes. These results show that the model has limited discrimination in some classes and needs improvement.

## 8. Conclusions

This study presented important and innovative findings using a combination of laser reflectance data and visual data for fish freshness classification. Laser reflectance measurements supported the analysis of physical changes on the surfaces of fish through optical reflectance properties. The experiments showed that laser reflectance data have limited predictive capacity, and their predictive performance demonstrated their role in providing complementary information. On the other hand, the stronger representational capability of the image data resulted in high accuracy rates, especially in classifications with models such as ConvNeXt and ResNet50. These results suggest that visual attributes are an important source of data for tasks such as fish freshness prediction.

The combination of laser data and visual data further improved the prediction performance, and it was observed that these two data types provide complementary information. While the combination of ResNet50 features and the MLP provided the highest accuracy, the integration of laser and visual data was found to be a promising method for more complex and high-accuracy tasks. However, the low accuracy rates observed in some classes, especially in the Day 2 class, suggest that there is still work to be done for more effective feature selection and model refinement.

In future studies, the generalization performance of the models can be improved by expanding the dataset with visual and laser reflection data obtained from different fish species. The current system has only been tested on mackerel; however, the proposed methodology can be easily adapted to different fish species with appropriate retraining and calibration processes. Due to differences in characteristics such as the skin structure, mucus density, and surface texture between species, retraining with species-based datasets is important. Additionally, integrating biological factors such as the fish’s age, size, and gender into the model can contribute to the more accurate and biologically consistent modeling of spoilage tendencies. In this study, only a laser sensor operating at a wavelength of 940 nm was used. However, future experiments using different wavelengths could provide a more detailed understanding of the tissue and optical responses. Furthermore, the integration of attention mechanisms could enable models to focus on the most meaningful regions within image and reflection data, thereby further improving their classification accuracy.

Finally, the sensor infrastructure used in this study is based on low-cost and portable components. This will enable real-time fish freshness monitoring to be performed in the future through smartphones or handheld compact devices. These applications have widespread and effective potential for use in fish markets, supermarkets, logistics chains, and end consumer points, both in terms of quality control and food safety.

## Figures and Tables

**Table 1:** Distribution of matched image and laser samples used in the dataset over three days.

Class	Number of Images	Number of Laser Samples
Day 1	258	258
Day 2	256	256
Day 3	256	256
Total	770	770

**Table 2:** Evaluation metrics.

Name	Description	Formula
Accuracy	Ratio of correctly classified samples to total samples	(TP + TN)/(TP + FP + FN + TN)
Precision	Ratio of correctly predicted positive samples to total predicted positive samples	TP/(TP + FP)
Recall	Ratio of correctly predicted positive samples to all actual positive samples	TP/(TP + FN)
F1-Score	Harmonic mean of precision and recall metrics	(2 × Recall × Precision)/ (Recall + Precision)

**Table 3:** Hyperparameter settings used for classifiers.

Classifier	Configuration Parameters
SVM	Kernel: RBF
RF	Number of Trees: 100
MLP	Hidden Layers: (100, 50) Max Iterations: 1000

**Table 4:** Performance of classifiers using only laser reflectance data.

Classifier	Acc ± Std (%)	F1-Score ± Std (%)
SVM	67.66 ± 3.84	67.24 ± 3.47
RF	**69.22 ± 3.25**	**68.35 ± 3.10**
MLP	67.79 ± 2.89	67.52 ± 2.71

**Table 5:** Classification performance using visual features extracted from CNN models.

Features	Classifier	Acc ± Std (%)	F1-Score (±Std) (%)
EfficientNet	SVM	83.12 ± 2.82	83.77 ± 2.91
	**RF**	**83.38 ± 1.34**	**84.11 ± 1.28**
	MLP	81.43 ± 1.46	81.64 ± 1.53
ConvNeXt	**SVM**	**85.97 ± 2.74**	**84.56 ± 2.65**
	RF	82.47 ± 3.94	78.87 ± 3.79
	MLP	86.49 ± 1.50	84.02 ± 1.43
MobileNet	**SVM**	**82.99 ± 3.57**	**80.20 ± 3.34**
	RF	78.44 ± 3.45	78.00 ± 3.28
	MLP	82.08 ± 1.20	79.90 ± 1.14
ResNet50	SVM	83.90 ± 1.99	82.12 ± 1.87
	RF	82.08 ± 2.48	79.93 ± 2.35
	**MLP**	**84.29 ± 2.77**	**83.22 ± 2.64**

**Table 6:** Performance of classifiers using fused visual and laser features.

Features	Classifier	Acc ± Std (%)	F1-Score (±Std) (%)
EfficientNet + Laser	**SVM**	**86.75 ± 2.45**	**87.42 ± 2.27**
	RF	80.91 ± 2.74	79.37 ± 2.65
	MLP	86.10 ± 2.31	86.10 ± 2.19
ConvNeXt + Laser	**SVM**	**88.31 ± 2.93**	**87.35 ± 2.78**
	RF	78.96 ± 4.49	76.01 ± 4.33
	MLP	87.53 ± 4.20	86.72 ± 4.07
MobileNet + Laser	SVM	83.25 ± 3.37	80.61 ± 3.19
	RF	77.53 ± 5.59	77.30 ± 5.43
	**MLP**	**85.71 ± 2.36**	**85.72 ± 2.21**
ResNet50 + Laser	SVM	85.71 ± 2.72	83.25 ± 2.59
	RF	81.30 ± 4.56	79.05 ± 4.38
	**MLP**	**88.44 ± 1.26**	**84.92 ± 1.17**

## Data Availability

The raw data supporting the conclusions of this article will be made available by the authors on request.
